# Genetic Analysis of Population Structure and Reproductive Mode of the Termite *Reticulitermes chinensis* Snyder

**DOI:** 10.1371/journal.pone.0069070

**Published:** 2013-07-22

**Authors:** Qiuying Huang, Ganghua Li, Claudia Husseneder, Chaoliang Lei

**Affiliations:** 1 Hubei Insect Resources Utilization and Sustainable Pest Management Key Laboratory, Huazhong Agricultural University, Wuhan, China; 2 Department of Entomology, Louisiana State University Agricultural Center, Baton Rouge, Louisiana, United States of America; University of Helsinki, Finland

## Abstract

The subterranean termite *Reticulitermes chinensis* Snyder is an important pest of trees and buildings in China. Here, we characterized genetic structure and reproductive modes of *R. chinensis* from China for the first time. A total of 1,875 workers from 75 collection sites in Huanggang, Changsha and Chongqing cities were genotyped at eight microsatellite loci. Analysis of genetic clusters showed two subpopulations in Chongqing city. The Huanggang population showed a uniform genetic pattern and was separated from the other populations by the largest genetic distances (*F*_ST_: 0.17–0.20). In contrast, smaller genetic distances (*F*_ST_: 0.05–0.12) separated Changsha, Chongqing-1 and Chongqing-2 populations. Chongqing-1 was the only population showing a genetic bottleneck. Isolation by distance among colonies in the Huanggang population indicated limited alate dispersal or colony budding. Lack of isolation by distance among colonies within the populations of Changsha, Chongqing-1 and Chongqing-2, suggested long-range dispersal by alates and/or human-mediated transport. Overall, extended family colonies (73.91%) were predominant in all four populations, followed by simple (20.29%), and mixed family colonies (5.80%). Most simple families were headed by inbred related reproductive pairs in the Changsha population, while most simple families in the Chongqing-1 population were headed by outbred unrelated pairs. Simple families in the Huanggang population were a mixture of colonies headed by outbred or inbred reproductive pairs. The sample size of simple families in the Chongqing-2 population was too small to yield significant results. Extended families in all four populations were headed on the average by ≤10 neotenics. Mixed families likely originated from pleometrosis. Presence of heterozygote genotypes showed that all neotenic reproductives collected in addition from five field colonies in Wuhan city were sexually produced, suggesting that these colonies did not undergo parthenogenesis. This study contributes to better understanding of the variance of genetic structure and reproductive mode in the genus *Reticulitermes*.

## Introduction

An increasing number of studies have employed molecular markers, such as microsatellites, to investigate population genetic structure and colony breeding system in termites, which has improved our understanding of evolutionary genetics, phylogeography, invasion biology, patterns and processes of dispersal, and social organization in termites [1], [2]. The analyses of genetic differentiation and patterns of gene flow within and among termite populations promoted understanding of population dynamics and dispersal patterns [3]–[5], and can help clarify the effect of ecological factors and human disturbance on the levels of genetic differentiation and gene flow [6]–[8]. Moreover, the studies on population genetic structure of invasive termites are helpful to identify potential source populations and to assess whether changes in genetic variability and/or breeding system are associated with invasiveness [9]–[11]. Previous studies on colony breeding structure showed that most populations of subterranean termites were composed of different percentages of simple and extended families [4], [12]–[16], while mixed families were generally less common [6], [17]. Additionally, the number of reproductives and the degree of relatedness among them in termite colonies can also be inferred using molecular markers [3],[18]–[22]. Recent studies on the modes of reproduction using microsatellite genotyping found that queens of *Reticulitermes speratus* and *R. virginicus* can use conditional parthenogenesis, where primary queens produce secondary queens by parthenogenesis (asexual queen succession AQS), but use sexual reproduction to produce workers [23], [24]. Studies on the model of conditional use of sexual and asexual reproduction are helpful to understand the advantages and disadvantages of different reproductive modes in termites [25]. However, it is still unknown how widespread AQS is among *Reticulitermes* spp.

The genus *Reticulitermes* has received much attention among the Isoptera when it comes to population and colony genetic studies, but almost all the species used in these studies were from America and Europe. In Asia, only colony genetic structure of *R. speratus* from Japan was studied using a small number of colonies on a limited spatial scale [26], but population genetic structure of this species has not yet been investigated. Since phylogeographic divisions were found among species of *Reticulitermes* termites across different countries even within Europe [27]–[30], it is necessary to thoroughly investigate and compare population genetic structure and colony breeding system of several representative termite species from different places in Asia. In a phylogenetic analysis of the family Rhinotermitidae, Austin *et al*. [31] found that *R. speratus* and *R. chinensis* were close relatives within the genus *Reticulitermes*, belonging to the same clade. Since *R. speratus* is one of the species featuring AQS, knowledge of the evolution of this unusual breeding strategy could be significantly advanced by studying how widespread AQS is within the genus *Reticulitermes* and if the development of AQS is congruent with phylogeny. Thus, we test the hypothesis that colonies of *R. chinensis* undergo AQS as well.

The subterranean termite *R. chinensis* is widely distributed in China, including Beijing, Tianjin, Shanxi and the Yangtze River drainage basin [32]. This termite species is an important pest of forest trees and urban buildings. To date, some basic information on the biology of *R. chinensis* has been obtained through field observations and laboratory experiments, including the growth of incipient colonies, the formation of neotenics, and the time of alate swarms [33], [34]. Since *R. chinensis* has cryptic foraging and nesting habits with hidden royal chambers, it is very difficult to find and collect reproductives. Thus, population genetic structures and colony breeding systems of *R. chinensis* are still poorly understood. The objectives of this study were to use microsatellite genotyping to (1) describe the population genetic structure of *R. chinensis*, (2) infer colony breeding system, (3) and determine whether colonies of *R. chinensis* undergo AQS as it has been suggested for other *Reticulitermes* species.

## Results

### Colony Assignments and Population Genetic Structure

**Figure 1:**
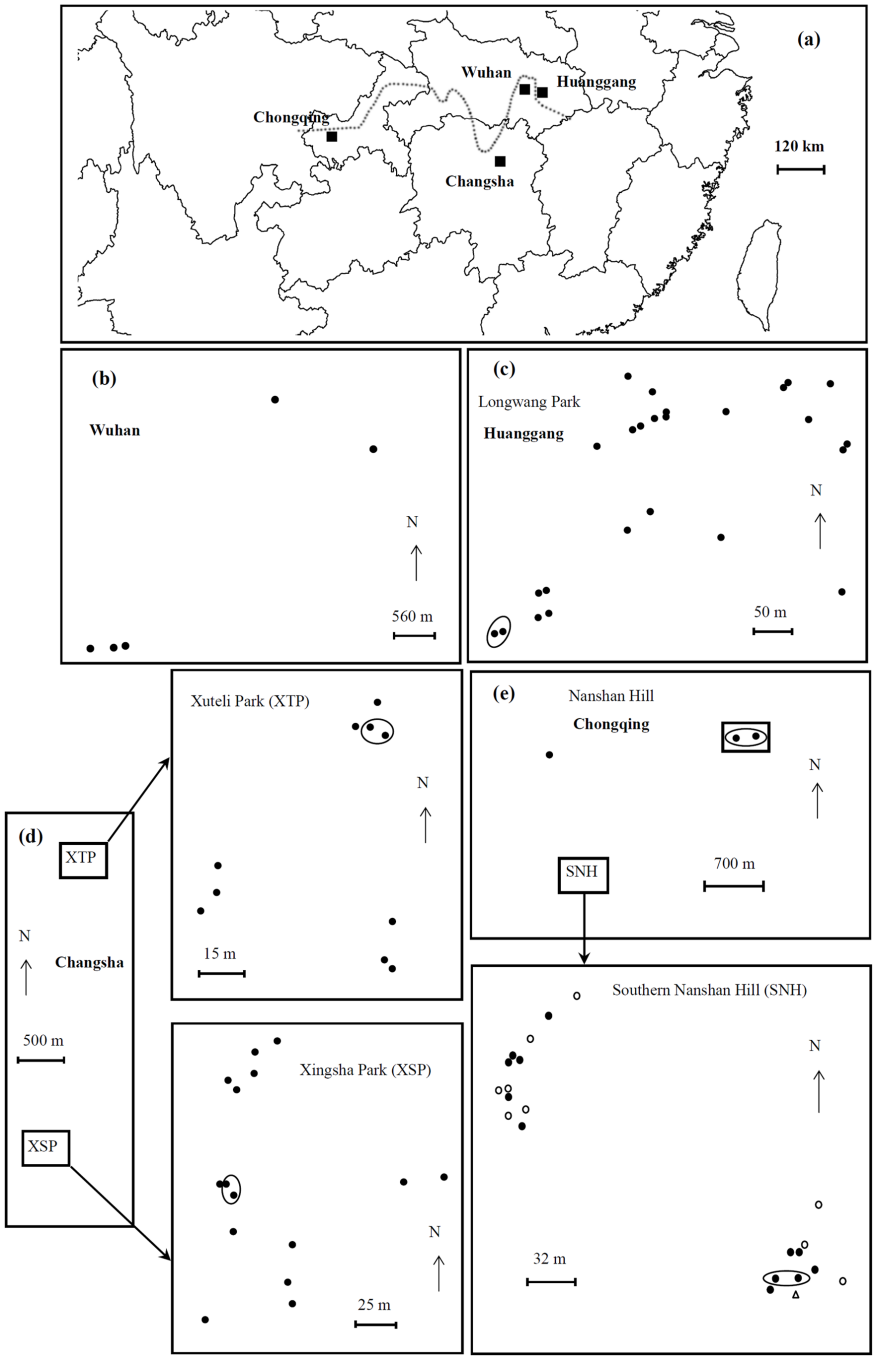
Map of the sample locations for the studies of population structure and reproductive mode. (a) Overview over the locations of the cities in China where samples were obtained from (Huanggang, Changsha and Chongqing and Wuhan). (b) Sample sites of entire nests including neotenics from Wuhan City, Hubei Province. (c) Sample sites of workers from Longwang Park,Huanggang City, Hubei Province. (d) Sample sites of workers from Xuteli Park and Xingsha Park, Changsha City, Hunan Province. (e) Sample sites of workers from Nanshan Hill, Nanan District, Chongqing City. The dotted line represents the Yangtze River. Genetic analyses revealed two subpopulations in Chongqing city (*filled circle* subpopulation 1; *open circle* subpopulation 2; *Open triangle* representing one colony that could not be assigned to either subpopulation of Chongqing. The loops represent samples assigned to the same colony.

**Figure 2:**
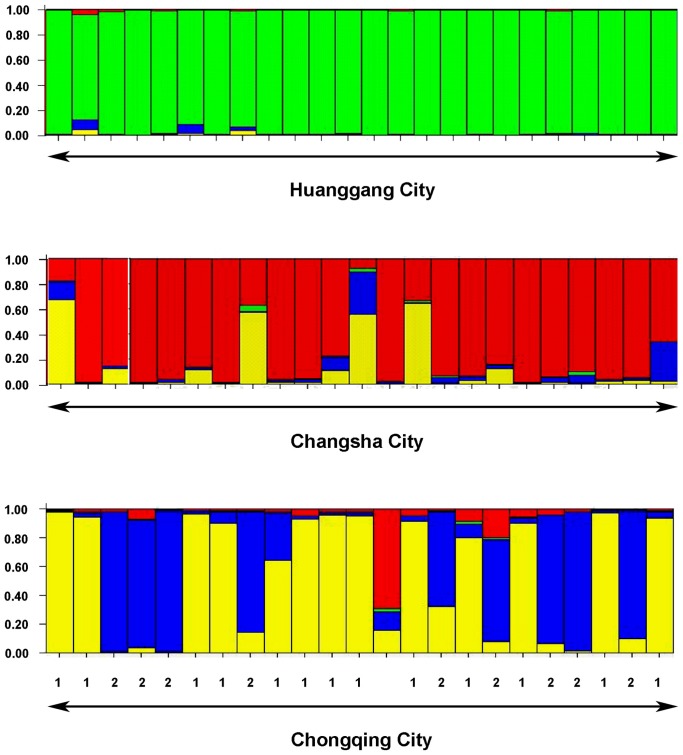
Assignment of individuals (each representing a colony) sampled from three cities to genetic clusters. Columns represent colonies, each color represent a different genetic cluster defined by STRUCTURE (*K* = 4). The colors in each column represent the likelihood with which a colony is assigned to each genetic cluster. The numbers (1 and 2) in this figure represent the colonies assigned to the Chongqing-1 population and the Chongqing-2 population, respectively.

**Figure 3:**
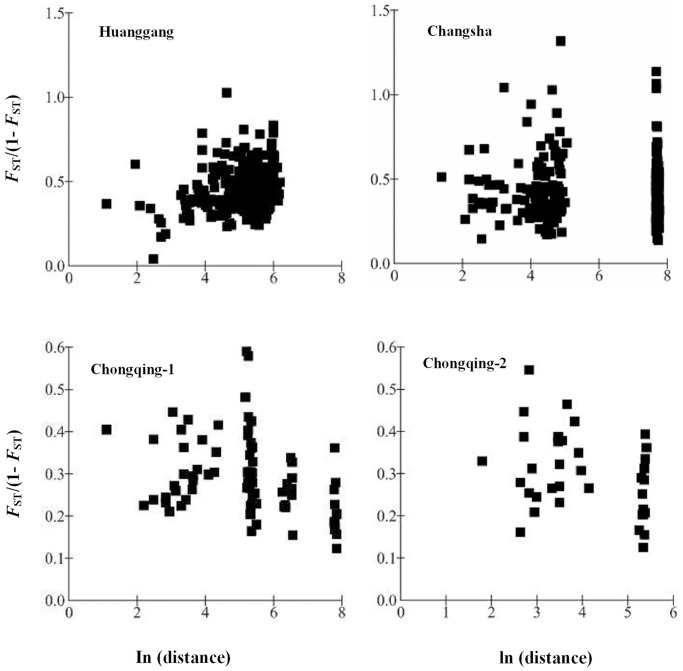
Isolation by distance analysis for the colonies within each population. The correlation coefficient was significant for the relationship for Huanggang population (*r* = 0.2245, *P* = 0.0014, Mantel test). The correlation coefficients were not significant for either of the relationships (*r* = 0.0130, −0.3284 and −0.3426, *P* = 0.4462, 0.9654 and 0.9015, Mantel test, for Changsha, Chongqing-1 and Chongqing-2 population, respectively).

STRUCTURE analysis showed that the *R. chinensis* colonies from Huanggang, Changsha and Chonqing cities (Figure 1) belong to two major genetic clusters (K = 2, DeltaK = 1348.8) but the Evanno plot (Figure S1) also shows the presence of a secondary pattern consisting of four genetic clusters indicating that there might be subpopulations in one of the regions (DeltaK = 417.5). The 25 samples collected from Huanggang (Figure 1c) belonged to 24 different colonies. No subpopulations were detected in Huanggang (Figure 2), but significant isolation by distance at 500 m spatial scale was found (Figure 3). The 25 samples collected from Changsha (Figure 1d) belonged to 23 colonies. STRUCTURE indicated the potential presence of multiple subpopulations (2–4 clusters) among the samples from Changsha (Figure 2). However, assignment of most colonies to any one of the clusters was weak (<80%). Clusters were not congruent with the two geographic clusters of sample sites (Figure 1d) and no significant isolation by distance was detected (Figure 3). Therefore, we considered all Changsha colonies to belong to one population in the further analyses. The 25 samples collected from Chongqing (Figure 1e) belonged to 23 colonies. Results generated from STRUCTURE indicated the presence of two distinct genetic clusters in Chongqing, with 13 colonies assigned to the first subpopulation (Chongqing-1) and 9 colonies assigned to a second subpopulation (Chongqing-2). One colony could not be assigned to either of the two subpopulations of Chongqing (Figure 1e, open triangle) but belonged to the genetic cluster representing the Changsha population (Figure 2). The majority (83%) of colonies were strongly (≥80%) assigned to one or the other subpopulation. Genetic separation was supported by 10 private alleles distinguishing subpopulation Chongqing-1 from Chongqing-2 and 15 private alleles distinguishing Chongqing-2 from Chongqing-1. Thus, colonies from the Chongqing-1 and Chongqing-2 subpopulations were treated as separate populations in the following analyses, thereby increasing the total number of populations to four. There was no significant isolation by distance within either subpopulation (Figure 3). Interestingly, there was no obvious relationship between the genetic clusters and geographical location of the colonies (Figure 1e). Evidence was found for a recent genetic bottleneck in the Chongqing-1 subpopulation, but not in Chongqing-2. The Chongqing-1 subpopulation had significant heterozygote excess under the infinite allele model and the two-phase model (*P* = 0.002) and marginal heterozygote excess under the stepwise mutation model (*P* = 0.097).

Permutation tests showed significant genetic differentiation among all four populations (P = 0.001, 6000 permutations) confirming the existence of four genetically differentiated groups indicated by STRUCTURE analysis. Genetic distances (*F*_ST_) among the four populations of *R. chinensis* ranged from 0.05 to 0.2 (Table S1). The Huangang population was clearly separated from the other populations by large genetic distances ranging from 0.17 to 0.20. Its genetic pattern was uniform showing little contributions to the genetic make-up of the other populations in the dataset. Although the remaining three populations were significantly differentiated from each other according to the permutation tests, the genetic distances ranging from 0.05 to 0.12 between the populations, were rather small in particular between the two Chongqing subpopulations (Table S1). Two colonies from Changsha were assigned to the genetic clusters representing the two Chongqing subpopulations with >80%. One colony from Chongqing could not be assigned to either subpopulation of Chongqing but could be assigned with 70% to the genetic cluster representing the Changsha population (Figure 2). The STRUCTURE results showing two main genetic clusters (Figure S1) confirm the separation of the Huangang population from the other three populations (Figure S2).

Table 1 shows the summary statistics for genetic variability and inbreeding in the populations. Between eight (*RS78*) and 21 (*Rf21-1*) alleles were detected per locus with a mean of 14.38 per locus. The expected heterozygosity ranged from 0.69 to 0.79, and the observed heterozygosity ranged from 0.55 to 0.84 (Table 1). The inbreeding coefficient ranged from −0.1 to 0.21. Overall, the four populations deviated significantly from Hardy–Weinberg equilibrium. There was a deficit of heterozygotes at three loci in the Huanggang population, and at two loci in the Changsha population. This heterozygote deficit at a subset of loci might have been caused by amplification failure of null alleles. Micro-Checker software confirmed deviations from Hardy-Weinberg-Equilibrium due to heterozygote deficit and suggested the occurrence of null alleles at locus *Rf21-1* (frequency: 0.31), *RS15* (frequency: 0.15) and *RS68* (frequency: 0.11) in the Huanggang population and at locus *Rf21-1* (frequency: 0.11) and *RS78* (frequency: 0.28) in the Changsha population. Heterozygote excess was detected across all loci in Chongqing-1 possibly due to a recent genetic bottleneck (see above). No evidence for linkage disequilibrium, large allele dropout or scoring error due to stuttering was detected in any of the four populations.

**Table 1:** Summary statistics for the four genetically differentiated populations.

Population	N	*N*_A_	*H*_E_	*H*_O_	*F*_IS_
Huanggang	24	9	0.79	0.65	0.19
Changsha	23	7.5	0.69	0.55	0.21
Chongqing-1	13	5.88	0.76	0.84	−0.1
Chongqing-2	9	5.88	0.75	0.65	0.13

*N*, number of colonies screened per location; *N*_A,_ average number of alleles per locus; *H*_E,_ expected heterozygosity; *H*_O,_ observed heterozygosity; *F*_IS,_ inbreeding coefficient.

### Colony Genetic Structure and Breeding System

Overall, extended family colonies were predominant (55–83%) in the four populations from Huanggang, Changsha and Chongqing, followed by simple family colonies (17–31%), and mixed family colonies (0–9%). The proportion of extended, simple and mixed family colonies varied among the four populations, with the highest proportion of extended families (83%) in the Huanggang population, the highest proportion of simple families (31%) in the Chongquing-1 population and the most mixed family colonies (9%) in the Chongquing-2 population (Table 2).

**Table 2:** *F*-statistics and relatedness coefficients for workers from *R. chinensis* colonies from four populations in China and expected for possible breeding systems of subterranean termite colonies previously derived from computer simulations [20], [21].

	*F*_IT_ (*SE*)	*F*_CT_ (*SE*)	*F*_IC_ (*SE*)	*r* (*SE)*
**Huanggang**				
Simple family colonies (n = 4)	0.14 (0.10)^a^	0.33 (0.05)	−0.29 (0.06)^a^	0.58 (0.05)^a^
Extended family colonies (n = 20)	0.18 (0.08)^A^	0.30 (0.02)	−0.19 (0.09)^A^	0.52 (0.02)^A^
**Changsha**				
Simple family colonies (n = 4)	0.23 (0.06)^b^	0.36 (0.05)	−0.20 (0.02)^b^	0.58 (0.05)^a^
Extended family colonies (n = 18)	0.14 (0.09)^C^	0.29 (0.02)	−0.21 (0.11)^AB^	0.50 (0.03)^A^
Mixed family colonies (n = 1)				
**Chongqing-1**				
Simple family colonies (n = 4)	−0.07 (0.06)^c^	0.19 (0.03)	−0.31 (0.07)^a^	0.40 (0.07)^a^
Extended family colonies (n = 8)	0.03 (0.04)^D^	0.25 (0.03)	−0.28 (0.04)^C^	0.48 (0.04)^A^
Mixed family colonies (n = 1)				
**Chongqing-2**				
Simple family colonies (n = 2)	0.03 (0.10)^a^	0.29 (0.07)	−0.35 (0.09)^a^	0.56 (0.10)^a^
Extended family colonies (n = 5)	0.11 (0.10)^A^	0.23 (0.02)	−0.16 (0.12)^AB^	0.42 (0.05)^A^
Mixed family colonies (n = 2)	−0.06 (0.04)^α^	0.11 (0.03)	−0.19 (0.04)^α^	0.24 (0.06)^α^
**Simulated breeding systems**				
(A) Simple family colonies with				
(1) outbred unrelated pairs	0^ac^	0.25	−0.33^a^	0.5^a^
(2) inbred related pairs	0.33^ab^	0.42	−0.14^ab^	0.62^a^
(B) Extended family colonies with inbreeding among multiple neotenics				
(1) *N*_f_ = *N*_m* = *_1, *X = *1	0.26^AC^	0.65	−0.14^A^	0.55^A^
(2) *N*_f_ = 2, *N*_m* = *_1, *X = *3	0.52^B^	0.59	−0.17^A^	0.78^B^
(3) *N*_f_ = *N*_m = _10, *X = *1	0.33^A^	0.34	−0.01^A^	0.51^A^
(4) *N*_f_ = 200, *N*_m = _100, *X = *3	0.33^A^	0.34	0^B^	0.5^A^
(C) Mixing between unrelated colonies, *N_f_* = *N_m_* = 1, *X* = 3, *p* = 0.8	0.57^β^	0.43	0.25^α^	0.55^α^
(D) Mixing between related colonies, *Nf = Nm = *1, *X = *3, *p = *0.9	0.66^β^	0.64	0.04^α^	0.77^α^
(E) Pleometrosis				
(1) colonies headed by two queens and one king	0^α^	0.19	–0.23^α^	0.38^α^
(2) colonies headed by two queens and one king, then *N*_f_ = *N*_m_ = 10, *X* = 3	0.27^α^	0.29	–0.03^α^	0.45^α^
(3) colonies headed by five queens and five kings, then *N*_f_ = *N*_m_ = 10, *X* = 3	0.1^α^	0.12	–0.02^α^	0.22^α^

*n*, number of colonies; *SE*, standard error derived from jackknifing over colonies (or loci if sample size ≤3). One sample *t-tests* were performed using *F*_IT_, *F*_IC_, and *r* values across individual colonies. Significant differences between empirical values and expected values are indicated by different letters (uppercase for extended families, lowercase for simple families, Greek alphabet lower case for mixed families). For simulated breeding systems, *X* represents generation number of neotenics within a colony; *N*_f_ and *N*_m_ represent number of replacement females and males per generation respectively; *p* presents mixing proportion between workers from different colonies.

The *F*-statistics and relatedness values estimated from the worker genotypes in each population are shown in Table 2, along with values derived from simulations for different breeding systems computed in previous studies [20], [21], [35]. Mean observed *F*_IT_ and *F*_IC_ values of simple family colonies in Changsha were significantly higher than the expected values in outbred unrelated pairs (Table 2, case A1) (all *P*<0.05, one sample *t* test), but not significantly different from the expected *F*_IT_ and *F*_IC_ values for inbred related pairs (Table 2, case A2). Thus, most of simple-family colonies in Changsha were headed by monogamous pairs of inbred related reproductives. However, mean observed *F*_IT_ and *F*_IC_ values of simple family colonies in Chongqing-1 were significantly and marginally significantly lower than the expected values in inbred related pairs, respectively (Table 2, case A2) (*P*<0.01, one sample *t* test), but not significantly different from the expected *F*_IT_ and *F*_IC_ values for outbred unrelated pairs (Table 2, case A1). Therefore, the majority of simple-family colonies in Chongqing-1 were likely headed by monogamous pairs of outbred unrelated reproductives. The empirical values for simple families in Chongqing-2 also matched closest with the assumption of outbred unrelated founder pairs, however, no statistical significances could be achieved due to the small sample size (n = 2). In Huanggang, the *F*-statistics and relatedness values of simple family colonies were not significantly different from either of the expected values and the standard errors were large (Table 2, cases A1 and 2). This indicates that some colonies in the Huanggang population might be headed by outbred unrelated pairs and others by inbred related pairs.

The values of *F*_IT_, *F*_IC_, and *r* of the extended family colonies in the Huanggang population were consistent with those expected for colonies having small numbers (≤10) of neotenic reproductives representing the first generation of replacement reproductives (Table 2, cases B1 and 3), but differed significantly (*P*<0.05, one sample *t* test) from breeding systems assuming three generations of replacement reproductives and/or hundreds of neotenics in at least one of the three genetic parameters (Table 2, cases B2 and 4). In Changsha, the extended family colonies showed *F*_IT_, *F*_IC_, and *r* values consistent with those expected for colonies headed by a pair of first generation neotenic reproductives (Table 2, case B1), but differed significantly (*P*<0.05, one sample *t* test) from the other breeding systems in at least one of the three genetic parameters (Table 2, cases B2, 3 and 4). In Chongqing-1, the values of *F*_IT_, *F*_IC_, and *r* for the extended family colonies differed significantly (*P*<0.05, one sample *t* test) from all the four simulated breeding systems in at least one of the three genetic parameters (Table 2, cases B1, 2, 3 and 4), thus no single predominant type (in regard to numbers and generations of neotenics) could be assigned to the extended families in this population. In Chongqing-2, the *F*_IT_, *F*_IC_, and *r* values in extended family colonies were consistent with the simulated values for colonies headed by the first generation of ≤10 neotenics (Table 2, cases B1, and 3), but differed significantly (*P*<0.05, one sample *t* test) from the other breeding systems with multiple generations and/or high numbers of neotenics in at least one of the three genetic parameters (Table 2, case B2 and 4).

The Changsha and Chongqing-1 populations contained only one mixed family colony each, so *F*-statistics could not be obtained. The *F*-statistics and relatedness estimates for the two mixed family colonies from Chongqing-2 were consistent with simulated values for pleometrosis (Table 2, case E1, 2 and 3), but *F*_IT_ differed significantly (*P*<0.05, one sample *t* test) from the breeding systems involving mixing of colonies (Table 2, cases C and D).

### Inferring Reproductive Mode of Neotenics’ Parents

Of the nine field colonies collected in Wuhan city (Figure 1a) and dissected to obtain the reproductives, four colonies contained single pairs of primary reproductives and were thus simple families. Five colonies (Figure 1b) contained multiple neotenics with a range of 1 to 51 secondary kings and 5 to 74 secondary queens (Table 3). Most of the secondary reproductives developed from nymphs, but there were one male and 5 female ergatoid neotenics (i.e. derived from workers) in colony 5, and two female ergatoid neotenics in colony 2 (Table S2). Neither primary kings nor primary queens were found in addition to the neotenics. We determined that all of the 59 secondary queens and 29 secondary kings genotyped were sexually produced because they were heterozygote at one to eight loci (Table S3). This allows us to test whether these neotenics originated from simple, extended or mixed family colonies. The results showed that the neotenics in colony 1, 3, 4 and 5 had more genotypes than possible for offspring of a monogamous pair or the observed frequencies of the genotypes deviated significantly from those expected in simple families (*P*<0.05, G-test). Thus, the neotenics from these four colonies were offspring of extended family colonies headed by multiple neotenics and as such arose at least two generations after colony foundation (Table 3). However, neotenics in colony 2 had six alleles at locus *Rf21-1*, which suggests that they originated from a mixed family colony (Table 3).

**Table 3:** Compositions of the five colonies headed by neotenics and reproductive mode of neotenics’ parents.

Colony	Location	Collectiondate	No. of secondary queens genotyped/collected	No. of secondary kings genotyped/collected	Reproductive mode of neotenics’ parents	Breeding system of the colonies of the neotenics’ parents
1	Shizi Hill, Wuhan City, China	1 Aug 2011	20/30	3/5	Sexual reproduction	Extended family
2	Shizi Hill, Wuhan City, China	15 Aug 2011	20/74	20/51	Sexual reproduction	Mixed family
3	Shizi Hill, Wuhan City, China	23 Apr 2011	8/8	4/4	Sexual reproduction	Extended family
4	Houshan Hill, WuhanCity, China	20 Apr 2011	6/6	1/1	Sexual reproduction	Extended family
5	Yujia Hill, Wuhan City, China	20 Apr 2011	5/5	1/1	Sexual reproduction	Extended family

## Discussion

Large-scale population genetic structure analysis revealed the existence of four populations with significant genetic differentiation in the dataset. The four populations were divided into two main genetic clusters. The population from Huanggang was separated from the other populations by comparatively large genetic distances and showed a uniform genetic pattern, suggesting that there was little contribution of alleles to or from the other populations. The sample sites from Huanggang lie north of the Yangtze River and are thus separated from the sample sites of Changsha and Chongqing, which are located south of the Yangtze River. Thus, geographical barriers, including the Yangtze River in addition to long distances, might limit gene flow between Huanggang and the other three populations. In contrast, the remaining three populations from Changsha, Chongqing-1 and -2 showed some level of gene flow among them. Surprisingly, colonies from Changsha were being assigned to the genetic clusters representing Chongqing populations and *vice versa*, which is indirect evidence for gene flow even across large distances (approx. 640 km). The most likely routes of migrants connecting Changsha and Chongqing are occasional human-mediated movement of infested material from one population to the other through roads and trade network, such as building timbers, ornamental plants, and barges travelling along rivers. Similarly, human transport was considered to be a factor in the gene flow for introduced *R. flavipes* populations between North American and France [9], for *R. urbis* between Balkans and Western Europe [8], and for *Coptotermes formosanus* between Fukue and Kyushu islands [7].

The existence of two populations with slightly different genetic characteristics in the same area in Chongqing was peculiar. The Chongqing-1 population showed signs of a genetic bottleneck, but the Chongqing-2 population was in mutation-drift equilibrium. This difference between the two populations colonizing the same area in Chongqing might be explained by the introduction history. The first introduction to Nanshan Hill in Chongqing took place in the early 1990s when a number of ornamental plants and landscape timbers were transported to Nanshan Hill for constructing Nanshan Arboretum [36]. The second introduction to Nanshan Hill in Chongqing happened in the early 2000s when a lot of building timbers and ornamental plants were transported to construct hotels in Nanshan Hill. To explain the difference between the two populations, we suggest that the earlier introduced population (Chongqing-2) might have contained a larger population size mitigating the initial bottleneck effects and/or enough time since the introduction has passed for the bottleneck to become undetectable, while the more recent introduction (Chongqing-1) still is experiencing the effects of a bottleneck [11]. Relative excess of heterozygosity is a transient effect and is expected to be detectable only for a limited number of generations [37]. Interestingly, bottlenecks could be detected as far back as the 1950’s and 60′s in another subterranean termite with a known introduction history, *C. formosanus* [11]. Either *R. chinensis* has a higher generation rate accelerating the return to mutation-drift equilibrium, the initial bottlenecks were not as severe as in *C. formosanus’* intercontinental journey (i.e. the initial founder population size was larger), and/or the small sample size prevented detection of weak bottleneck effects in the Chongqing-1 population [38]. Since there are no conceivable barriers preventing gene flow, we expect the differences between the sympatric populations of Chonqing-1 and -2 to disappear over time.

There was significant isolation by distance among colonies in the Huanggang population, indicating that dispersal by primary reproductives is relatively limited over the spatial scale studied (550 m), either because of short range mating flights by alates and/or frequent colony reproduction by budding. Similarly, significant isolation by distance has been found in two *R. flavipes* populations from France [9]. In contrast, there was no significant isolation by distance among colonies in the Chongqing-1 and Chongqing-2 populations, at the spatial scale studied (about 2.6 km). This lack of isolation by distance suggests that during mating flights reproductives disperse relatively far over the spatial scale studied. Simple-family colonies in Chongqing-1 were headed by monogamous pairs of outbred unrelated reproductives, which further supports the hypothesis of long-range dispersal flights of alates. In addition, the human-mediated introduction and movement of colonies via landscaping and building material and activities might have further masked any isolation by distance imposed by natural dispersal limits.

Similar to the Chongqing populations, the colonies in the Changsha population also did not show isolation by distance at the spatial scale studied (2.4 km). In contrast to the Chongqing colonies, however, simple family colonies in Changsha were headed by inbred related monogamous pairs. This result does not support the existence of long-range mating flights in this population. Alternatively, as suggested for the Chongqing population, human-mediated colony movement may play an important role in population expansion and reducing population viscosity, i.e. isolation by distance, in Changsha.

The four populations of *R. chinensis* in our study consisted of a combination of simple family and extended family colonies. A few mixed family colonies were found in all but the Huanggang population. Overall, extended families with a low number of neotenics and generation turnovers were the dominant breeding system. This finding is consistent with previous studies in *R. ﬂavipes* [9], [13], [21], [39], *Zootermopsis nevadensis nuttingi* [40], *R. grassei* [35], [41], *R. urbis* [8] and *C. formosanus* in Guangdong, Hunan, Kauai, Maui 1 and Charleston [10], [11], [16]. Moreover, small percentages of mixed family colonies were also detected for *R. ﬂavipes* [13], [39] and *R. grassei* [41]. Proportions of termite colonies with different breeding systems vary across populations and depend on age structure of the colonies, dynamics of colony-colony interactions, food quantity and quality, soil characteristics and disturbance or treatment [4], [14], [20], [21], [42]. There were plenty of 20–30 years old pine trees and camphor trees in the parks and hills of sample collection in Huanggang, Changsha and Chongqing, suggesting that the *R. chinensis* colonies had enough time and food to grow and develop into extended families headed by neotenics [35]. Even more recently established populations, such as the Chongqing populations, showed predominance of extended family colonies, possibly due to disturbance and movement of colonies by human activities, which might lead to splitting colonies, death and replacement of colony founders and an overall increase in extended family colonies in the population as was previously shown in a population of another subterranean termite, *C. formosanus*, which was subjected to disturbance by building and landscaping activities [42].

Simple family colonies in Changsha were headed by inbred related monogamous pairs. This result suggested that dispersal by primary reproductives is limited in the Changsha population as discussed above. Similarly, simple family colonies headed by inbred related monogamous pairs were found in studies of *R. grassei* in southwestern France [35], *Z. n. nevadensis* in North Carolina [40], and *C. formosanus* in Kyushu, Fukue and Hawaii [7], [11], [16]. However, simple family colonies in Chongqing-1 were headed by outbred unrelated monogamous pairs, which suggested that colonies reproduce by relatively long-range mating flights within this population. Similarly, simple family colonies headed by outbred unrelated monogamous pairs were reported in studies of other termite species, including *R. flavipes* in Massachusetts [21] and North Carolina [3], [13], [43], [44], [45], *R. virginicus* in North Carolina [44], and *C. formosanus* in Charleston [10]. Moreover, our results indicated that simple family colonies in Huanggang were a mixture of colonies whose workers were offspring of outbred and unrelated or inbred and related monogamous pairs. A previous study in one *R. grassei* population in southwestern France has also reported that simple family colonies were a mixture of colonies headed by outbred or inbred monogamous pairs [35]. The sample size of only two simple families in the Chongqing-2 population was too small to reject either of the simulated breeding systems with sufficient statistical support.

The strongly negative *F*_IC_ values for the extended families in Huanggang and Changsha suggest that the mean number of neotenics in our colonies was low (≤10), and that all neotenics were inbred and ultimately the descendants from monogamous pairs of founders. Similarly, extended family colonies were found to be headed by low numbers of inbred neotenics in populations of *R. ﬂavipes* [3], [43], [44], [45], *R. hageni* [45], *R. virginicus* [45], *C. formosanus* in South Carolina, Louisiana, North Carolina, Hawaii, Guangdong and Hunan [10], , *Z. n. nevadensis* [40], *Z. angusticollis* and *Z. n. nuttingi* [5]. Surprisingly, the extended family colonies in Chongqing-1 were inconsistent with all of the simulated breeding systems. Similar results have been found in studies of other termite species and were suggested to arise from colonies with multiple reproductives representing a mixture of several breeding systems [35], [40], [43]. Although classified as extended family colonies, the breeding structure estimates for extended family colonies in Chongqing-1 were most similar to values expected for simple family colonies headed by outbred monogamous pairs. One possibility is that these colonies only recently made the transition from simple to extended family by developing neotenics and thus contained still a measurable proportion of offspring of the previous (i.e. simple family) generation [40], [42]. Since colonies of the Chongqing-1 population were probably recently moved by human activities (see above), it is plausible to assume that disturbance might have increased incidental death of the colony founders resulting in recent development of replacement reproductives and thus the transition to extended family colonies [42]. Overall, the results suggested that the extended family colonies in Chongqing-1 contained small numbers of neotenics as shown for the populations of Huanggang and Changsha. The values of *F*_IT_, *F*_IC_, and *r* for extended families in Chongqing-2 were not significantly different from those for the simulated breeding system based on high numbers of neotenics (Table 2, Case B4), which is possibly due to the low sample size of extended family colonies in this population. Nevertheless, the strongly negative *F*_IC_ values (−0.16) for the extended families in Chongqing-2 suggested that the number of neotenics in these colonies was low (≤10). Low numbers of neotenics were also found in the extended family colonies of the other three populations (Huanggang, Changsha and Chongqing-1).

Mixed family colonies in Chongqing-2 displayed breeding structure estimates consistent with pleometrosis. Evidence for mixed family colonies originating from pleometrosis was previously found in the lower termite *Z. nevadensis* [40] and the higher termites *Nasutitermes corniger* and *M. michaelseni* [46], [47]. In pleometrosis, multiple queens and kings (alates) cooperate in colony foundation and expansion during the primary stages of colony development [18], [47]. However, pleometrosis has never been found in any of the other *Reticulitermes* species reported from America and Europe [1]. Some other factors have been suggested to explain mixed family colonies, including colony fusion, invasion of mature colonies by other alates, or sharing foraging galleries by neighboring colonies [40], [43]. For example, mixed family colonies were the result of colony fusions in native and introduced populations of *R. ﬂavipes* [43], [48]. Since the percentage of mixed families was low in *R. chinensis* populations, larger sample sizes are needed to unequivocally determine the origin of mixed family colonies.

In addition to information about breeding systems in *R. chinensis* colonies derived from worker genotypes, we also directly observed number and types of reproductives in nine field colonies collected from Wuhan. Four of the dissected field colonies were simple families headed by a pair of primary kings and queens, and five colonies contained multiple neotenics. Three of the five colonies had low numbers of reproductives (6–12). The range is largely consistent with the results from the genetic analyses of worker genotypes from the populations of Huanggang, Changsha and Chongqing. The percentage of our collected field colonies containing secondary kings in *R. chinensis* was higher than those in *R. speratus* and *R. virginicus* [23], [24]. In *R. speratus,* secondary kings have been found in only 6.67 percent of colonies (2 of 30 colonies; [23]). Secondary kings were not found at all in five colonies of *R. virginicus* [24]. In contrast, each colony of *R. chinensis* in the present study had at least one secondary king and up to 51 secondary kings in colony 2. Almost all the neotenics in *R. speratus* and *R. virginicus* differentiated from nymphs [23], [24], but there were eight ergatoid neotenics in *R. chinensis* in this study. Our results based on the inferred genotypes of the neotenics’ parents in each of the five colonies showed that the preceding parental generation in the five colonies also consisted of multiple reproductives. The breeding system of the five colonies headed by the neotenics’ parents was in four cases an extended family colony headed by neotenics and in one case a mixed family colony. These results suggest that extended family colonies headed by neotenics and mixed family colonies can continue the colony life cycle by producing the next generation of neotenics in *R. chinensis*.

Our results showed that all the neotenics in the five field colonies of *R. chinensis* were sexually produced, suggesting that these colonies did not undergo conditional parthenogenesis. Based on the classical model of AQS [23], we did not expect the ergatoid neotenics to be produced parthenogenetically since they are derived from workers, which are almost exclusively sexually produced in other *Reticulitermes* species that undergo AQS [24]. We also did not necessarily expect the secondary kings to be parthenogens. Queens usually increase their reproductive output and fitness by producing multiple replacement daughters via parthenogenesis, which at least in *R. speratus* can continue the queen’s full genetic contribution throughout generations by producing parthenogenetic daughters themselves. However, none of the nymphoid secondary queens in *R. chinensis* was homozygote at all loci.

In contrast to our findings in *R. chinensis*, the seven colonies of *R. speratus* in Kyoto [23] and the five colonies of *R. virginicus* in North Carolina and Texas [24] have been shown to undergo AQS. A previous phylogenetic analysis of the family Rhinotermitidae found that *R. speratus* and *R. virginicus* belonged to distinctly different clades and were thus not closely related, which prompted the hypothesis that AQS might have arisen early in the evolution of the genus *Reticulitermes*. However, the same study showed that *R. chinensis* is a close relative of *R. speratus* with both species sharing the same clade [31]. Nevertheless their reproductive mode differs since *R. chinensis* apparently does not undergo AQS. Therefore, AQS has either evolved independently in *R. speratus* and *R. virginicus* [24] or *R. chinensis* secondarily lost the ability to undergo AQS.

Clinal variation of ecological factors, such as temperature, moisture, seasonality, availability of wood and soil composition have been shown to influence breeding structure in *Reticulitermes* species [49], however, geographic parthenogenesis has not yet been studied in termites. In facultative parthenogenic plants and animals in general, asexual populations tend to occur at higher latitudes and altitudes and in disturbed habitats [50]. Previous studies in populations of the stick insect, *Clitarchus hookeri* showed that the incidence of parthenogenesis increased at higher latitudes both in the Northern and Southern Hemisphere [51]. The colonies of *R. chinensis* in our study were collected from lower latitudes than the colonies of *R. speratus* and *R. virginicus* (>35°N) that exhibited parthenogenesis, except for one *R. virginicus* colony from South Houston (<30°N) [23], [24]. Thus, it would be interesting to study whether *R. chinensis* colonies located further north and at higher altitudes than the ones in this study display AQS or if parthenogenesis simply does not occur in this species regardless of the local ecological conditions.

## Materials and Methods

### Sampling for Population Genetic Study

Between May and July 2010, samples of *R. chinensis* were collected from fallen trees and stumps from 75 collection sites in three geographic regions of China (Figure 1a): Longwang Park, Huanggang city, Hubei Province (Figure 1c); Xuteli Park and Xingsha Park, Changsha city, Hunan Province (Figure 1d); and Nanshan Hill, Nanan District, Chongqing city (Figure 1e). All the parks and hills of sample collection were inside cities. The geographic distance was 300 km, 650 and 800 km between Huanggang and Changsha, between Changsha and Chongqing, and between Chongqing and Huanggang, respectively. The latitudes ranging from 28°14′20.1″ to 30°27′34.5″ and longitudes ranging from 106°36′53.8″ to 114°52′00.3″ of all sample sites were determined using a GPS localizer (Table S4). More than 30 workers per sample were collected in the field and immediately preserved in 95% ethanol. A few soldiers per sample were also collected in order to identify the species.

None of the parks and hills where samples were collected are privately-owned or protected in any way, and *R. chinensis* is not endangered or protected. Thus, no specific permissions were required to access and sample at these locations.

### Microsatellite Genotyping

DNA was extracted from whole worker bodies using the DNeasy Tissue Kit (Beijing Dingguo Changsheng Biotech Co. Ltd). Twenty-five workers from each collection site were genotyped at 8 microsatellite loci (Table S5), identified from *R. flavipes* [52], [53]. PCR amplifications were performed as described in Vargo [52]. PCR products were separated by electrophoresis on 6% polyacrylamide gels run on a LI-COR 4300 DNA analyzer. Alleles were scored using the computer program SAGA^GT^ (LI-COR, Inc.). The software Micro-Checker [54] was used to test loci for null alleles and possible scoring errors derived from large allele dropout and the presence of microsatellite stutter bands.

### Colony Identification

Samples from the 25 collection sites in each region were tested for significant genotypic differentiation using log-likelihood *G*-Statistics (FSTAT, [55]). *P* values were obtained by permutations of the multilocus genotypes between each pair of samples and standard Bonferroni corrections were used. If samples significantly differed from each other, they were assigned to different colonies [11], [14].

### Population Genetic Structure

Since colony members are close kin, only a single randomly chosen individual per colony was used for population genetic analyses to avoid bias by nonindependent genotypes. The number of genetic clusters in the three geographic regions was assessed on the basis of a comparison of the penalized log likelihoods over independent simulation runs using STRUCTURE 2.3.3 with variable numbers of assumed genetic clusters (*K*) [56]. All simulations were done using the default settings, i.e. the admixture model and correlated allele frequencies, with 100,000 runs in the data collection phase following a burn-in period of 100,000 runs [11]. Three repeats of simulations were run for each model with different numbers of assumed genetic clusters (K = 1 to 7). The number of genetic clusters was determined following the Delta K method of Evanno *et al*. [56] using STRUCTURE HARVESTER v.0.6 [57]. According to the methods of Pritchard *et al*. [58], the estimated membership coefficients in K clusters (degree of admixture) of each multilocus genotype (i.e. individual representing a colony) were determined and were graphically displayed in form of a histogram. Colonies from the three geographical regions were probabilistically assigned to the populations for which they showed dominant membership (>80%). As long as considerable structure was found, datasets would be separated into subsets and reanalyzed until the number of subpopulations was determined. Confirmed subpopulations were treated as genetically differentiated populations. Pairwise genetic differentiation among populations at the 1/1000 nominal level was confirmed via permutation tests using multilocus G-statistics with Bonferroni corrections as implemented in FSTAT [55] to obtain the final number of populations in the data set.

To estimate isolation by distance, pairwise *F*_ST_ values for all pairs of colonies within each population were obtained. The matrix correlation between *F*_ST_/(1−*F*_ST_) and the ln of geographic distance (m) for all pairs of colonies within each population was determined [10]. The significance of the correlation coefficient was estimated using Mantel tests (one sided *p*-values from 10,000 permutations, TFPGA v. 1.3, [59]. In addition, genetic differentiation (*F*_ST_) between each pair of populations was computed based on a hierarchical analysis using the program GENETIC DATA ANALYSIS version 1.1 (GDA, [60]). Exact tests for Hardy–Weinberg equilibrium for each locus and linkage disequilibrium between all pairs of loci were performed using the program GDA (GDA, [60]) with 3200 shufflings [7]. To determine whether populations had experienced a recent genetic bottleneck, worker genotypes were tested for heterozygosity excess across loci based on a Wilcoxon sign-rank test under three mutation models (infinite allele model, two-phased model of mutation, and stepwise mutation model) using the program Bottleneck v. 1.2.02 [37].

### Colony Genetic Structure and Breeding System

Referring to the previous methods [20]–[21], [43], colonies were classified into one of three family types: simple, extended or mixed families. Colonies would be regarded as simple families if the numbers of genotypes of the workers were compatible with those expected for offspring of a monogamous pair and if the frequencies of the observed genotypes were not different from the expected Mendelian ratios. Colonies would be regarded as extended families if there were more than four genotypes, or if genotype frequencies were significantly different from expected Mendelian ratios (*P*<0.05, G-test). Mixed family colonies would be characterized by the presence of more than four alleles at one or more loci.

Colony genetic structure was analyzed by *F*-statistics using the methods of Weir & Cockerham [61] as implemented in FSTAT [55]. Separate analyses were performed for each population to assure that *F* values (*F*_IT,_ *F*_CT_ and *F*_IC_) used to infer breeding system were not confounded by higher-level genetic structure [7], [20], [21]. The coefficient of relatedness (*r*) was determined by the average nest mate relatedness within all colonies of the respective population using the program FSTAT [55]. These empirical values were then compared to expected values resulting from previously compiled computer simulations of possible breeding systems of termites via one-sample t-tests not assuming equal variances (P<0.05), to infer predominant breeding systems in the populations of this study [20], [21], [35].

### Inferring Reproductive Mode of Neotenics’ Parents

To infer reproductive mode of neotenics’ parents, that is, parthenogenesis versus sexual reproduction in *R. chinensis*, we used the methods of Matsuura *et al*. [23] and Vargo *et al*. [24] to collect field colonies and conduct microsatellite analysis. Between July 2010 and September 2011, we collected four field colonies with primary reproductives (Figure S3a) and five field colonies with neotenics (Figure S3b) in Wuhan city (Figure 1a). All reproductives from each colony were immediately preserved in 95% ethanol in a vial for subsequent microsatellite analysis. We discriminated secondary neotenic reproductives from primary reproductives based on the lighter body color and absence of eyes in neotenics. Sex of all reproductives was identified based on the conformation of the caudal sternites under a stereoscope [62]. According to the presence or absence of wing pads, neotenics would be divided into nymphoid and ergatoid neotenics [23].

We genotyped up to 20 male and female neotenics (nymphoid and ergatoid) from five field colonies collected at Shizi Hill (colony 1, 2 and 3), Houshan Hill (colony 4), and Yujia Hill (colony 5) in Wuhan city using the eight microsatellite loci and protocols described above (Table S3). The latitudes ranging from 28°28′49.86″ to 30°32′15.3″ and longitudes ranging from 114°20′58.38″ to 114°24′9.66″ of the five field colonies were also determined using a GPS localizer (Figure 1b). If neotenics were homozygous at all loci, they would be considered to be produced by parthenogenesis. If neotenics were heterozygous at one or more loci, they would be considered to be sexually produced [23], [24]. For sexually produced neotenics, we tested whether these neotenics are offspring of simple, extended or mixed family colonies using the same methods as described for workers above [20]–[21], [43].

## Supporting Information

Figure S1 Evanno plot derived from STRUCTURE HARVESTER for detecting number of genetic clusters.
(TIF)

Figure S2 Assignment of colonies to genetic clusters by STRUCTURE (K = 2).
(TIF)

Figure S3 Primary and secondary reproductives of *Reticulitermes chinensis* collected from field colonies in Wuhan City. (a), primary king (PK) and primary queen (PQ). (b), secondary kings (SK) and secondary queens (SQ).
(TIF)

Table S1 Genetic distance (*F*_ST_) among the four populations.
(DOC)

Table S2 Composition of secondary reproductives in the five field colonies.
(DOC)

Table S3 Genotypes of secondary queens (SQ) and secondary kings (SK) from the five colonies. S: Sexually produced. Genotypes marked ‘na’ were unavailable due to failed PCR reactions. Colony numbers are the same as those in Table 3.
(XLS)

Table S4 Latitudes and longitudes of sample sites from three geographic regions in China.
(XLS)

Table S5 Worker genotypes at the eight microsatellite loci from Huanggang, Changsha and Chongqing Cities. Genotypes marked ‘na’ were unavailable due to failed PCR reactions.
(XLS)
