# Supplementary figures and images for: Genetic Analysis of Population Structure and Reproductive Mode of the Termite Reticulitermes chinensis Snyder
Source: PLoS One. 2013 Jul 22;8(7):e69070. doi: 10.1371/journal.pone.0069070 (PMC3718804; doi:10.1371/journal.pone.0069070)

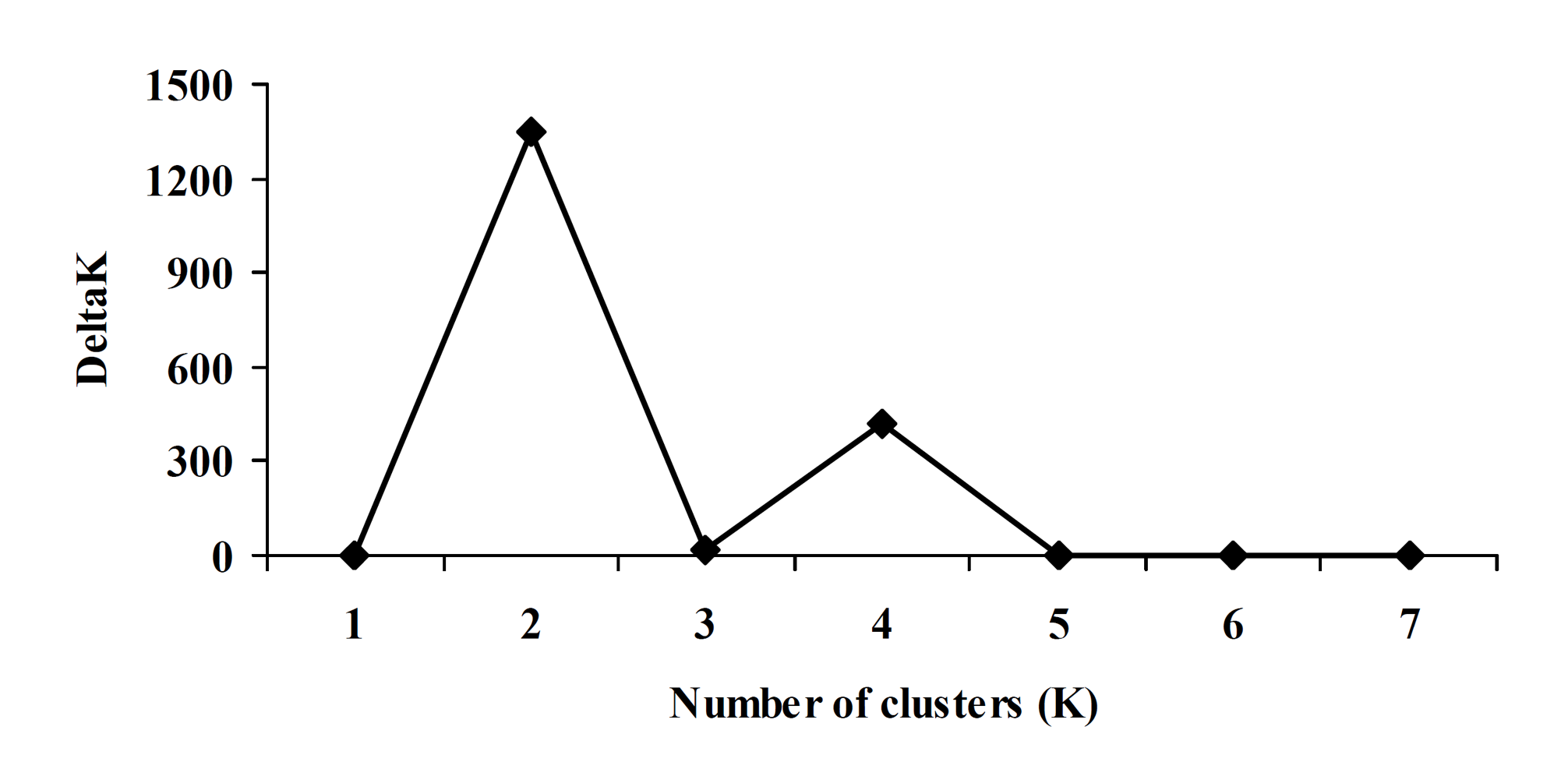

Supplement: Figure S1 — Evanno plot derived from STRUCTURE HARVESTER for detecting number of genetic clusters. (TIF) [file pone.0069070.s001.tif]

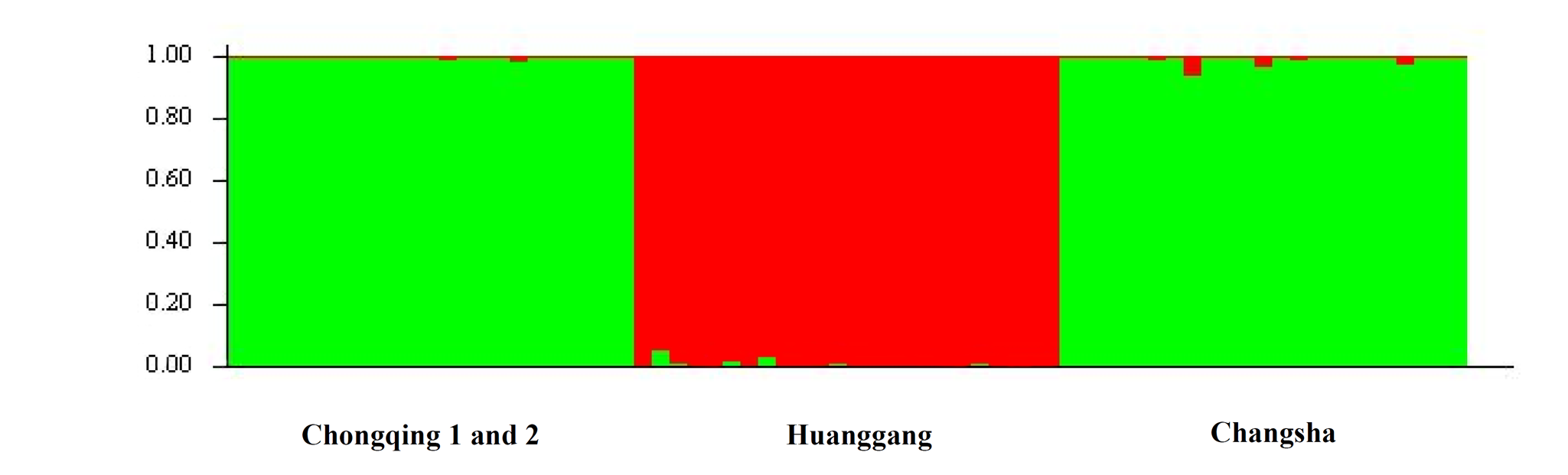

Supplement: Figure S2 — Assignment of colonies to genetic clusters by STRUCTURE (K = 2). (TIF) [file pone.0069070.s002.tif]

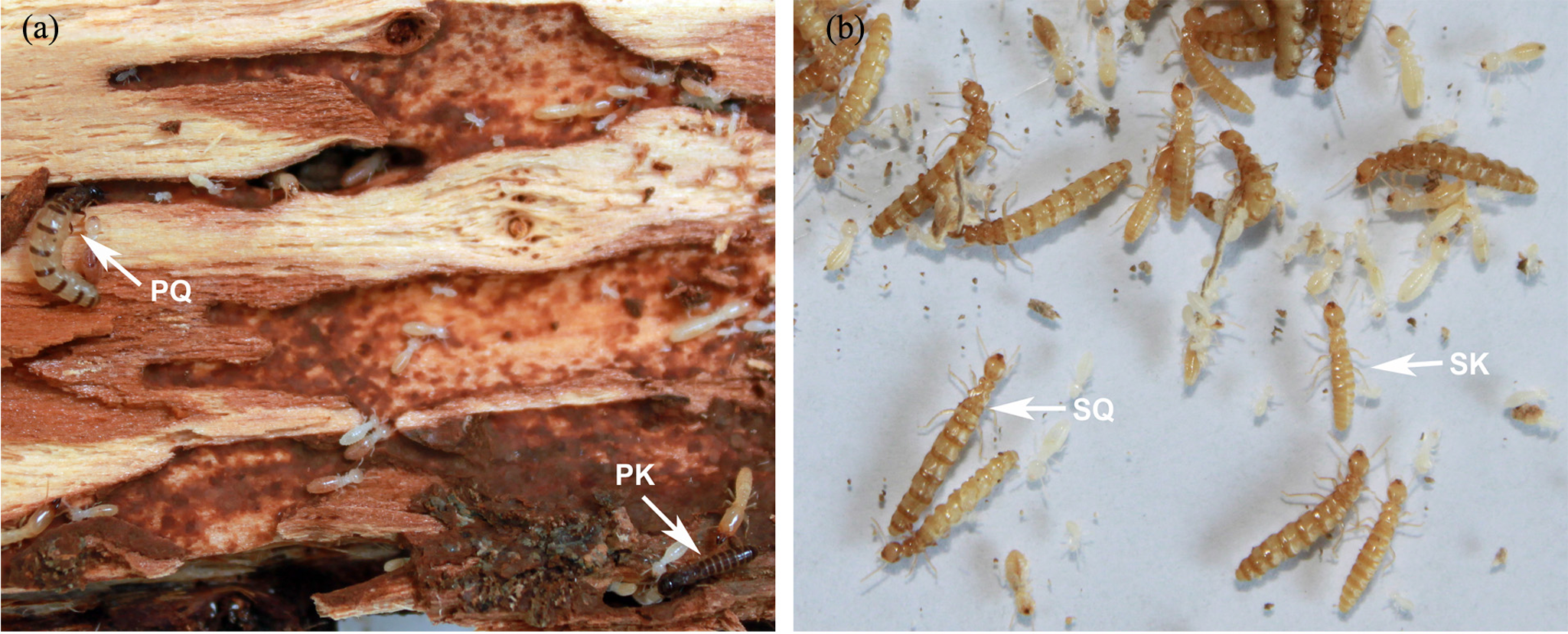

Supplement: Figure S3 — Primary and secondary reproductives of Reticulitermes chinensis collected from field colonies in Wuhan City. (a), primary king (PK) and primary queen (PQ). (b), secondary kings (SK) and secondary queens (SQ). (TIF) [file pone.0069070.s003.tif]
