# Supplementary material for: Genetic Analysis of Population Structure and Reproductive Mode of the Termite Reticulitermes chinensis Snyder
Source: PLoS One. 2013 Jul 22;8(7):e69070. doi: 10.1371/journal.pone.0069070 (PMC3718804; doi:10.1371/journal.pone.0069070)
Supplement: Table S1 — Genetic distance (F ST) among the four populations. (DOC) [file pone.0069070.s004.doc]

**Table S1** Genetic distance (*F*_ST_) among the four populations*.*

| Population | Huanggang | Changsha | Chongqing-1 | Chongqing-2 |
| --- | --- | --- | --- | --- |
| Huanggang | 0 | 0.20 | 0.17 | 0.18 |
| Changsha | 0.20 | 0 | 0.10 | 0.12 |
| Chongqing-1 | 0.17 | 0.10 | 0 | 0.05 |
| Chongqing-2 | 0.18 | 0.12 | 0.05 | 0 |
