# Supplementary material for: Genetic Analysis of Population Structure and Reproductive Mode of the Termite Reticulitermes chinensis Snyder
Source: PLoS One. 2013 Jul 22;8(7):e69070. doi: 10.1371/journal.pone.0069070 (PMC3718804; doi:10.1371/journal.pone.0069070)
Supplement: Table S2 — Composition of secondary reproductives in the five field colonies. (DOC) [file pone.0069070.s005.doc]

**Table S2 C**omposition of secondary reproductives in the five field colonies.

| Conoly code | Secondary kings | | |  | Secondary queens | | |
| --- | --- | --- | --- | --- | --- | --- | --- |
|  | Nymphoid | Ergatoid | Total |  | Nymphoid | Ergatoid | Total |
| 1 | 5 | 0 | 5 |  | 30 | 0 | 30 |
| 2 | 51 | 0 | 51 |  | 72 | 2 | 74 |
| 3 | 4 | 0 | 4 |  | 8 | 0 | 8 |
| 4 | 1 | 0 | 1 |  | 6 | 0 | 6 |
| 5 | 0 | 1 | 1 |  | 0 | 5 | 5 |
| Total | 61 | 1 | 62 |  | 116 | 7 | 123 |
